# REPLY BY THE AUTHORS: Comment on Polygamy, Sexual Behavior in a Population Under Risk for Prostate Cancer Diagnostic: An Observational Study From the Black Sea Region in Turkey

**DOI:** 10.1590/S1677-5538.IBJU.2020.0297

**Published:** 2020-07-31

**Authors:** Abdullah Cirakoglu, Erdal Benli, Ahmet Yuce

**Affiliations:** 1 Ordu University Faculty of Medicine Department of Urology Ordu Turkey Department of Urology, Ordu University, Faculty of Medicine, Ordu, Turkey

*To the editor,*

We thank Özkidik et al. for their interest and for their letter to the Editor (1). I will try to answer the questions they raised.

The authors do not think it is possible for all patients to have similar lifestyles and nutritional habits. It is only possible to ensure all conditions are equal with animal studies. In animal model studies, animals are housed in cages in the same place, fed with the same feed and water, creating an ideal environment. However, when working with humans, such an implementation does not comply with the reality of life. As stated in our paper, our region receives nearly no migration from other areas. Most people in the region have lived here for at least three generations. The culture, lifestyle and nutritional habits have been shaped by the climatic conditions, plant cover and geographic structure of the region. As this is not a place where people from different cultures migrate and live together like in metropolitan areas, the lifestyles and nutritional habits of people in the region are naturally similar. It is not possible to perform a study similar to animal models, but our region is close to ideal due to similar lifestyles and nutritional habits in the region.

The authors report it will be beneficial to state previous urethritis and prostatitis attacks. It should not be forgotten that urethritis and prostatitis are not diseases that always provide clinical findings. The PCPT study identified inflammation in 52.1% of samples from patients with biopsy performed (2). Similarly, some sexually transmitted diseases may be asymptomatic and therefore underreported (3). These infectious agents may maintain their presence latently especially in women. Similarly, they may not always cause clinical symptoms in males but may remain latent. As a result, the number of prostatitis and urethritis attacks patients suffered will not reflect the true infection rates. On the other hand, our study was not a study designed to reveal the cause-outcome relationship. It is an observational study. The results of our study identified that those with higher partner numbers had higher incidence of prostate cancer. The reason for this may be previous urethritis and prostatitis attacks, just as there may be other causes. It is necessary to design different studies to reveal the cause-outcome correlations about this topic.

There is no consensus in the literature about whether ejaculation frequency is effective on the development of prostate cancer or not. It is very difficult to determine a clear result about this topic. Prostate cancer development involves a long process. There are many factors affecting the frequency of ejaculation such as religious beliefs, environmental factors, health problems, etc. Similarly, even if people are married, their frequency of monthly sexual relations is variable. It is not possible to find definite numbers for this topic. All numbers given are subjective and there is always an error rate. To reveal clear information about this topic, it is necessary to monitor the study group for years, and record monthly ejaculation frequencies at certain intervals. In fact, even at the end of such a study, no definite evidence may be reached due to uncertainty about the effects of other factors. It is possible that the reason for uncertainty in the literature is due to this.

Additionally, as our study group was not monitored for years, it is not possible to clearly provide numbers about the monthly number of sexual relations for any year. As we state in the text, this data was obtained from patients when they came to the clinic. In our country, the beginning of regular sexual relations generally occurs with marriage. As the marital age of people is very variable, it is not possible to make an objective assessment by accepting a certain age limit. Additionally, the reliability of information given may be lower. The mean age in our patient group was nearly 65 years. When we asked these patients about their monthly sexual relations frequency from 20-30 years, from 30-40 years and from 40-50 years ago, their responses will have lower reliability. Information given about younger years and the present period will be subjective when answers are grouped according to age, but we think that responses when asked about relation frequency in youth and at present will be closer to the reality.
